# Supplementary material for: Erythrocyte sodium buffering capacity status correlates with self-reported salt intake in a population from Livingstone, Zambia
Source: PLoS One. 2022 Mar 2;17(3):e0264650. doi: 10.1371/journal.pone.0264650 (PMC8890657; doi:10.1371/journal.pone.0264650)
Supplement: S2 Table — (DOCX) [file pone.0264650.s002.docx]

**Erythrocyte sodium buffering capacity status correlates with self-reported salt intake in a population from Livingstone, Zambia**

Sepiso K. Masenga,^1^  Leta Pilic,^2^ Malani Malumani, ^1^ Benson M. Hamooya,^1^

^1^HAND Research Group, School of Medicine and Health Sciences, Mulungushi University, Livingstone campus, Zambia

^2^Faculty of Sport, Allied Health and Performance Science, St. Mary’s University, Twickenham, London, UK

**Emails:** Sepiso K. Masenga [smasenga@mu.ac.zm](mailto:smasenga@mu.ac.zm) – Leta Pilic [leta.pilic@stmarys.ac.uk](mailto:leta.pilic@stmarys.ac.uk) – Malani Malumani [drmalumani@yahoo.com](mailto:drmalumani@yahoo.com) - Benson M. Hamooya [benmalambo@gmail.com](mailto:benmalambo@gmail.com)

**Corresponding Author:**

Dr. Sepiso K. Masenga

Mulungushi University,

School of Medicine and Health Sciences

Department of Physiological Sciences,

HAND Research groups, Livingstone, Zambia

[sepisomasenga@gmail.com](mailto:sepisomasenga@gmail.com) or smasenga@mu.ac.zm

| **S2 Table 1. Multiple linear analysis of factors associated with erythrocyte sodium sensitivity including estimated salt intake from 24-hr urine samples** | | | | |
| --- | --- | --- | --- | --- |
| **Characteristic** | **beta** | **Standard error** | **p** | **95% CI** |
| **Female** | 10.29 | 15.33 | 0.50 | -20.51 – 41.10 |
| **Unemployed** | 8.80 | 13.86 | 0.52 | -19.05 – 36.66 |
| **Red blood cell count** | 0.66 | 4.08 | 0.87 | -7.54 – 8.88 |
| **Hemoglobin** | -3.08 | 3.73 | 0.41 | -10.59 – 4.43 |
| **Self-reported high salt intake** | 8.22 | 6.04 | 0.17 | -20.37 – 3.94 |
| **Estimated salt consumption** | 4.54 | 1.61 | **0.007** | 1.30 – 7.79 |
| *All factors statistically significant were added to the multilinear model | | | | |
